# The flavor and acceptability of six different potassium-enriched (sodium reduced) iodized salts: a single-blind, randomized, crossover design

**DOI:** 10.1186/s40885-016-0054-9

**Published:** 2016-10-17

**Authors:** Akram Maleki, Ali Reza Soltanian, Fatemeh Zeraati, Vida Sheikh, Jalal Poorolajal

**Affiliations:** 1 Department of Epidemiology, School of Public Health, Hamadan University of Medical Sciences, Hamadan, Iran; 2 Department of Biostatistics, School of Public Health, Hamadan University of Medical Sciences, Hamadan, Iran; 3 Noncommunicable Diseases Research Center, Hamadan University of Medical Sciences, Hamadan, Iran; 4 Department of Pharmacology, School of Pharmacy, Hamadan University of Medical Sciences, Hamadan, Iran; 5 Department of Internal Medicine, School of Medicine, Hamadan University of Medical Sciences, Hamadan, Iran; 6 Research Center for Health Sciences, Hamadan University of Medical Sciences, 65157838695 Hamadan, Iran

**Keywords:** Potassium chloride, Sodium chloride, Hypertension, Blood pressure, Cross-over studies

## Abstract

**Background:**

Hypertension is a serious public health problem. Potassium-enriched salt is suggested as a tool for lowering blood pressure. However, its flavor and taste acceptability is essential for population-based salt reduction strategy and needs to be well-understood. This trial assessed the flavor and taste acceptability of six different potassium-enriched iodized salts in the general population.

**Methods:**

We conducted this crossover trial from May to June 2016, enrolling 100 normal volunteer subjects aged 11 to 64 years. We compared regular sodium chloride salt (placebo) with six different potassium-enriched (sodium reduced) iodized salt (experiment), including 0 %, 5 %, 10 %, 15 %, 20 %, 25 %, and 30 %. The participants served as their own control and received a placebo and a sequence of the experiments. They tasted the two salts sequentially and stated their preference and acceptance. Each subject received all salts.

**Results:**

More than 80 % of participants who either did not distinguish between the two salts even in high potassium-enriched salts or preferred potassium-enriched salt (*P* < 0.001). The number of participants who preferred the flavor of potassium-enriched salt was greater than the number of subjects who preferred the flavor of regular sodium chloride.

**Conclusion:**

Our findings indicated that the six different potassium-enriched salts had a public acceptability of at least 80 % among normal subjects from the general population. Although the acceptability of the potassium-enriched salts by a more general population group would require to be confirmed, universal use of this salt may help us achieve the target of 30 % relative reduction in mean population intake of sodium by 2025.

## Background

Noncommunicable diseases (NCDs) are the leading causes of death, accounting for almost two thirds of all deaths globally [1]. Although easy to diagnose, hypertension is still called the “silent and invisible killer” that rarely causes symptom [2]. It is one of the most powerful independent predictors of major cardiovascular events and stroke [3, 4]. Almost 51 % of stroke and 45 % of ischemic heart disease deaths are attributable to high systolic blood pressure (BP) [5]. Hypertension is a serious public health problem. Evidence has shown that more than 30 % of those affected by hypertension are unaware of their condition, which is frequently only discovered in the event of serious life threatening complications [6]. Among several predictors of hypertension, salt reduction is an important, cost-effective intervention in lowering BP and reducing the global risk of cardiovascular disease [4, 7, 8].

WHO recommended the Global Action Plan, including nine voluntary global targets to achieve a 25 % relative reduction in premature mortality from NCDs by 2025. A 30 % relative reduction in mean population intake of salt/sodium is one the targets to be attained by 2025 [9]. A population intake of less than 5 g of salt or 2 g of sodium per person per day is recommended by WHO for the prevention of cardiovascular diseases. This target is achievable and safe for both adults and children [10].

Potassium-enriched salt is suggested as a means of lowering BP. It works in two ways: (a) reducing sodium chloride removes the BP-raising effect of sodium; and (b) increasing potassium chloride reduces BP by the blood pressure-lowering effect of potassium [7]. The results of a long-term randomized trial indicated that potassium-enriched salt reduced cardiovascular disease mortality and medical expenditures in elderly people [11].

Considering consumer acceptability when approaching sodium reduction [12]. Taste acceptability of the potassium-enriched salt is a question that should be answered. Community-based intervention programs will be successful only if they are universally accepted. Suitability of the flavor of the potassium-enriched salt is essential for population-based salt reduction strategy and needs to be well-understood. Therefore, we carried out this trial to assess the flavor and overall taste acceptability of six different potassium-enriched (sodium reduced) iodized salts in the general population.

## Methods

We conducted a single-blind, randomized, crossover trial in Hamadan and Arak cities, west of Iran, from May to June 2016. We received verbal informed consent from all parents. The Ethics Committee of Hamadan University of Medical Sciences approved the trial. The protocol was not registered with the Iranian Registry of Clinical Trials because the participants were selected from healthy people.

Phase I clinical trials usually involves 20 to 100 normal volunteer subjects [13]. Accordingly, we selected a maximum of 100 healthy volunteer subjects aged 11 to 64 years from the general population. Participants with taste impairment or known systemic diseases were excluded from the study.

**Fig. 1:**
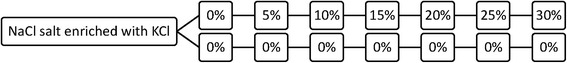
Crossover design for assigning the participants to the experiment group (receiving potassium-enriched ionized salt) versus control group (receiving regular sodium chloride ionized salt)

The planned crossover design is shown in Fig. 1. In this study, we compared regular sodium chloride iodized salt (placebo) with six different potassium enriched (sodium reduced) iodized salt (experiment), including 0 %, 5 %, 10 %, 15 %, 20 %, 25 %, and 30 %. In each period, participants randomly received the placebo and a sequence of experiments. Then, we asked the subjects to taste the two salts and state their preference and acceptance. If participants found out any difference between the two salts, we should indicate which one was more acceptable (less bitter and less spicy). Each subject served as his or her own control. All subjects participated for the same number of periods and received the same number of salts. Actually, each subject received all salts. Within each period, the participants compared every two salts simultaneously. There was a washout period of 5 to 10 min between the periods [14]. In washout periods, the subjects drank some water to remove the taste of the salt that they had already tested.

**Fig. 2:**
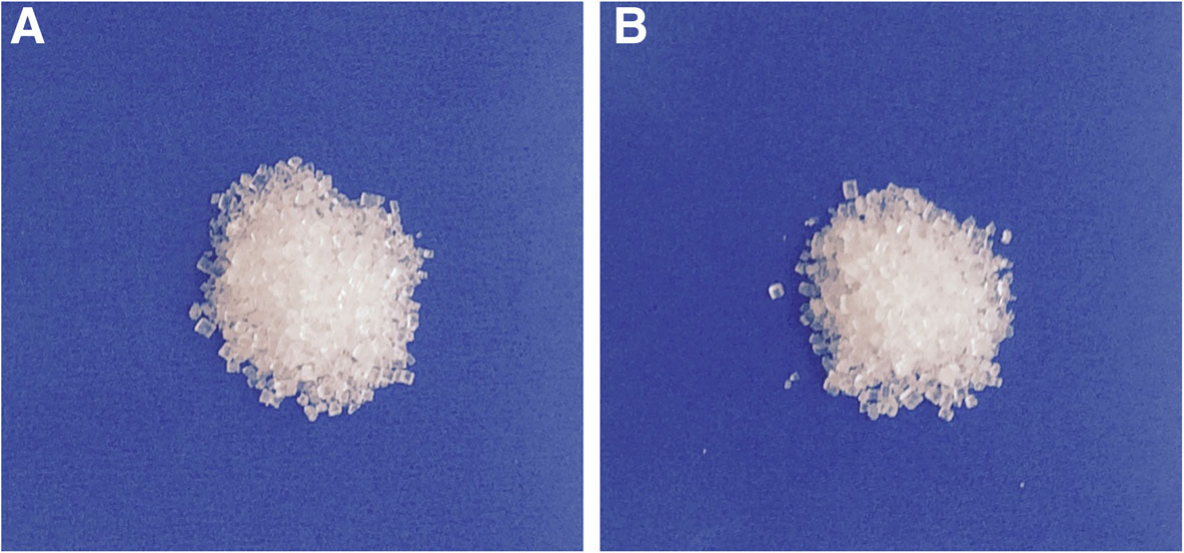
The delivery of regular sodium ionized salt (**a**) and potassium-enriched ionized salt (**b**) to the participants for tasting with similar smell and color

We set up a single-blind design. For this purpose, different concentrations of potassium-enriched salts were prepared by the Department of Pharmacy and labeled by a pharmacologist (F.Z.). In each period, we gave the participant equal amount of both salts with similar smell and color (Fig. 2). Therefore, the participants were unaware of the type of salts that they tasted.

We used the chi-squared test and Fisher exact test for analysis of categorical variables. All statistical analyses were performed at a significance level of 0.05, using Stata software, version 11 (StataCorp, College Station, TX, USA).

## Results

The characteristics of the study population are given in Table 1. Of the 100 volunteers (55 females and 45 males) 78 were nonsmokers, 14 were smokers, and 8 were ex-smokers. A majority of the participants had a college education. The mean (SD) age of the participants was 38.13 (10.70) years.

**Table 1:** Characteristics of the study population

Characteristics	Number (%), *n* = 100
Gender	
Female	55 (55.0)
Male	45 (45.0)
Smoking status	
Nonsmoker	78 (78.0)
Smoker	14 (14.0)
Ex-smoker	8 (8.0)
Educational level (yr)	
Primary school (1–5)	9 (9.0)
Secondary school (6–8)	8 (8.0)
High school (9–12)	29 (29.0)
College	54 (54.0)

The participants’ preference and acceptance about regular sodium chloride salt and potassium-enriched salt is given in Table 2. When the concentration of potassium was low, a majority of the participants did not make a distinction between the two types of salt. As the concentration of potassium increased the number of participants who could make a distinction between the two salts increased too. However, the number of participants who preferred potassium-enriched salt was greater than the number of participants who preferred regular sodium chloride salt in all concentrations but 10 %.

**Table 2:** Comparison of the flavor and acceptability of six different potassium-enriched iodized salts (KCl) versus regular sodium chloride iodized salt (NaCl)

KCL concentrations	Favors NaCl	Equal	Favors KCl	χ^2^	*P* value
0 %	-	96^a^	-	-	-
5 %	3	91	6	149.78	0.001
10 %	4	94	2	165.68	0.001
15 %	9	80	11	98.06	0.001
20 %	12	61	27	37.82	0.001
25 %	21	48	31	11.11	0.004
30 %	20	43	37	8.54	0.014

^a^4 participants reported difference between the two salts while they both were regular NaCl salt

We also compared the acceptability of regular sodium chloride iodized salt versus acceptability of six different potassium-enriched iodized salts or no discrimination between the flavors of the two salts. The results are given in Table 3. According to these results more than 80 % of participants either did not distinguish between the two salts even in high potassium-enriched salts or preferred potassium-enriched salt (*P* < 0.001).

**Table 3:** Acceptability of regular sodium chloride iodized salt (NaCl) versus acceptability of six different potassium-enriched iodized salts (KCl) or no discrimination between the flavor of the two salts using Fisher exact test (*P* < 0.001)

KCL concentrations	NaCl was accepted	KCl was accepted or no discrimination
5 %	3	97
10 %	4	96
15 %	9	91
20 %	12	88
25 %	21	79
30 %	20	80

## Discussion

Our findings indicated that all different potassium-enriched iodized salts are generally acceptable. A large-scale, blinded randomized trial conducted in rural northern China confirmed the overall acceptability of the saltiness and flavor of a salt substitute (65 % sodium chloride, 25 % potassium chloride and 10 % magnesium sulphate) [15]. An 11-day single-blind cross-over feeding trial showed that white bread in which 30 % of the sodium was replaced by potassium salts had acceptability scores similar to the standard bread [16]. Another study conducted by Kamleh et al. showed that a 30 % substitution of sodium chloride with potassium chloride in Akkawi cheese was acceptable [17]. On the other hand, an eight-week study conducted on 69 normotensive volunteers to test the feasibility and acceptability of two low-sodium (less than 70 mEq), high-potassium (greater than 100 mEq) diets reported that potassium enriched salt was unacceptable [18].

Beside its acceptability, befits and harms of the potassium-enriched salt intake at the population level needs to be investigated in large scale. Evidence based on randomized trial including potassium supplementation as high as 200 mmol/day for several weeks has shown no serious adverse effects [19]. Although serious adverse effects have not generally been reported, a few studies reported mild toxicity, especially gastrointestinal symptoms, from an extremely high potassium intake in supplement form [20–22]. However, according to the evidence from estimates of current potassium intakes in European countries, the probability of adverse effects from potassium intake from food sources (up to 5–6 g/day in adults) is considered to be low for the generally healthy population. In addition, long-term intakes of potassium supplements as high as 3 g/day, in addition to intake from foods, have been shown to be associated with no adverse effects [23].

An average potassium intake of 120 mmol/day has been recommended by the United States [24]. The WHO recommends a potassium intake of at least 90 mmol/day (3510 mg/day) from food for adults to reduce BP and risk of cardiovascular disease, stroke and coronary heart disease. The WHO also suggests a potassium intake of at least 90 mmol/day from food for children to control BP [25]. Evidence has shown that a 10-mmol increase in daily potassium intake can reduce the risk of stroke-associated mortality by 40 % (*P* < 0.001). This effect was independent of other dietary variables, including the intake of calories, fat, protein, fiber, calcium, magnesium, and alcohol [26]. The largest reduction in BP appears when potassium intake increases to 90–120 mmol/day. Intake above this dose does not seem to have any additional benefit [27].

Based on the Global Action Plan recommended by the WHO, a 30 % relative reduction in mean population intake of sodium is one the targets to be attained by 2025. Potassium-enriched salt has less sodium than regular salt. Therefore, in addition to the strategy of the reduction of salt to less than 5 g/day, intake of potassium-enriched (sodium reduced) salt can help us achieve this goal faster. Indeed, a combination of actions, that is, decrease of sodium and increase of potassium has more beneficial BP lowering effect than a change in either electrolyte on its own [28, 29].

The main limitation of this study was that we just evaluated the flavor and acceptability of various potassium-enriched salts. However, we are not aware of the harms that may be associated with long-term use of iodized potassium chloride at the population level. This issue needs further investigation.

## Conclusion

According to our findings, all different potassium-enriched iodized salts had a public acceptability of at least 80 % among normal subjects from the general population. Although the acceptability of the potassium-enriched salts by a more general population group would require to be confirmed, universal use of this salt may help us achieve the target of 30 % relative reduction in mean population intake of sodium by 2025.
